# Prevalence of Common Infectious Diseases After COVID-19 Vaccination and Easing of Pandemic Restrictions in Israel

**DOI:** 10.1001/jamanetworkopen.2021.46175

**Published:** 2022-02-01

**Authors:** Shimon Amar, Yonat Shemer Avni, Norm O’Rourke, Tal Michael

**Affiliations:** 1 Department of Family Medicine, Siaal Research Center for Family Medicine and Primary Care, Faculty of Health Sciences, Ben-Gurion University of the Negev, Be’er Sheva, Israel; 2 Clalit Health Services, Southern District, Be’er Sheva, Israel; 3 Clinical Virology Laboratory, Soroka University Medical Center, Faculty of Health Sciences, Ben-Gurion University of the Negev, Be’er Sheva, Israel; 4 Department of Public Health and Multidisciplinary Center for Research on Aging, Ben-Gurion University of the Negev, Be’er Sheva, Israel; 5 Department of Public Health, Ben-Gurion University of the Negev, Be’er Sheva, Israel

## Abstract

**Importance:**

Social restrictions intended to limit the transmission of SARS-CoV-2 may have also been associated with decreased rates of other communicable diseases. Evidence suggests that infection incidence rates (IRs) are rebounding after easing of social restrictions (eg, mask mandates). The reemergence of infectious disease complicates efforts to manage the ongoing COVID-19 pandemic.

**Objective:**

To examine IRs of frequently occurring infectious diseases after a successful SARS-CoV-2 vaccination campaign in Israel and cessation of social restrictions.

**Design, Setting, and Participants:**

This cross-sectional study was conducted using records for respiratory and gastrointestinal infectious diseases at 209 community clinics in southern Israel from 2017 to 2021. Included patients attended community clinics from January 1, 2017, to June 30, 2021.

**Exposures:**

Incidence of infectious diseases was estimated in the first 3 months after the easing of social restrictions (ie, April-June 2021) across age groups.

**Main Outcomes and Measures:**

Age-specific and disease-specific weekly IRs per 100 000 population for April to June were compared between 2017 and 2021 and expected current IR was estimated using segmented linear regression. Growth rates of respiratory infections across years and weekly diagnoses detected by real-time polymerase chain reaction testing were also compared.

**Results:**

Among 386 711 patients with a total of 1 221 568 visits to community clinics, the mean (SD) age was 27.29 (23.93) years, and there were 202 494 (52.3%) male patients and 184 217 (47.7%) female patients. Children aged 0 to 3 years had significantly increased rates of respiratory and gastrointestinal infection diagnoses (IR ratio, 2.64; 95% CI, 2:30-2.91; *P* < .001). In addition, incidence of non–SARS-CoV-2 respiratory infections were significantly increased across age groups (IR ratio, 1.74; 95% CI, 1.56-1.94; *P* < .001).

**Conclusions and Relevance:**

These morbidity trends observed in Israel suggest that similar trends could occur in coming months in other countries after easing of COVID-19–related restrictions, particularly with the ongoing challenges of SARS-CoV-2 variants.

## Key Points

**Question:**

Were COVID-19–related social restrictions in Israel associated with changes in the spread of certain infectious diseases across age groups?

**Findings:**

This cross-sectional study of 386 711 patients in community clinics in Israel found an increase in incidence rates of various infections among children aged 0 to 3 years and in respiratory infections among all age groups during 3 months after the easing of COVID-19–related social restrictions.

**Meaning:**

These findings suggest that as global COVID-19 vaccination rates increase and social restrictions are lifted, patterns of non–SARS-CoV-2 infection transmission observed late spring in Israel may be seen elsewhere, requiring early preparation.

## Teaser

This cross-sectional study examines changes in the incidence of respiratory infectious diseases after the easing of COVID-19 social restrictions in Israel.

## Introduction

**Figure 1.**
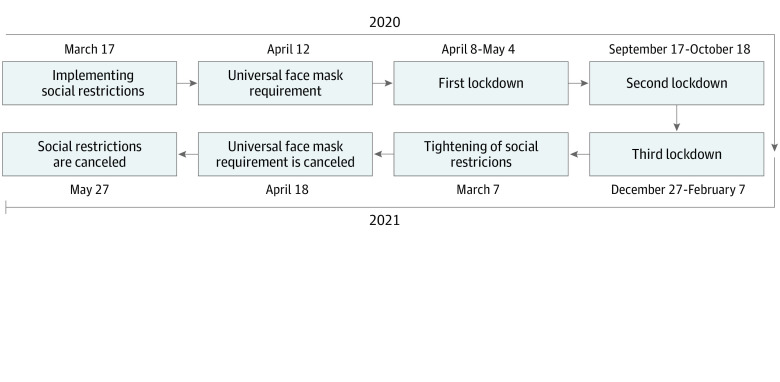
Timeline of Social and Behavioral Restrictions and Easing, 2020 to 2021

After a national campaign that vaccinated most adults in Israel against COVID-19 by March 25, 2021,^1^ the government began easing social restrictions, with a complete rescindment on April 18, although the indoor mask requirement was later reinstated (Figure 1). Social restrictions included nonpharmaceutical interventions (NPIs), such as shelter-in-place orders, universal masking, social distancing, and 3 national lockdowns, each intended to decrease the burden of COVID-19 morbidity and mortality. Additionally, these social restrictions were associated with decreases in non–SARS-CoV-2 infections, such as influenza and the common cold.^2^ There is growing global evidence suggesting that NPIs are associated with decreased rates of COVID-19 and non–COVID-19 infectious diseases, and thus a decreased burden associated with seasonal influenza, other upper and lower respiratory infections,^3^ and food-borne^4^ diseases.

However, after major declines in 2020,^3^ the incidence of respiratory infections is increasing in the Southern Hemisphere^5^ and the US.^6^ Studies describe a steep resurgence in respiratory syncytial virus (RSV) infections among infants, as reflected in increased rates of severe respiratory diseases in younger age groups. Agha et al^6^ posited that social distancing during the pandemic may have been associated with decreased exposure to RSV among neonates, and hence decreases in their subsequent immunity. To date, age-specific incidence rates (IRs) of non–COVID-19 common infectious diseases compared with rates in prepandemic years have not been reported, to our knowledge.

The increase of infectious disease rates along with the arrival of the delta variant of SARS CoV-2^7^ are of significant concern, given that already-strained health care systems may become overwhelmed. Now that North American and most European countries have immunized most citizens, social restrictions are being lifted. This suggests that patterns of non–COVID-19 communicable disease transmission observed in late 2021 spring in Israel may be seen in other Organization for Economic Cooperation and Development nations later this year.

Using data from community health clinics, we examined the epidemiological characteristics of community-acquired infections in April through June 2021 in southern Israel. Understanding these trends may potentially contribute to the development and implementation of rapid interventions to decrease the burden of disease on health care systems.

## Methods

This cross-sectional study was approved by the Clalit Health Services (CHS) Research Ethics Committee with a waiver of informed consent given the use of a deidentified data source. This study is reported following the Strengthening the Reporting of Observational Studies in Epidemiology (STROBE) reporting guideline for cross-sectional studies.

### Participant Data

We examined anonymized medical records of patients served by Israel’s largest health care organization (ie, CHS), which insures most of the population. Health care in Israel is universal, provided to all residents by 1 of 4 health care organizations, supported by member premiums and progressive income taxation.^8^ CHS data were extracted using the MDClone platform.

Inclusion criteria included all visits related to common infectious diseases by individuals of all ages at 209 clinics in southern Israel from January 1, 2017, to June 30, 2021. We then restricted our focus to visits resulting in *International Classification of Diseases, Ninth Revision* (*ICD-9*) diagnoses of respiratory or gastrointestinal community-acquired infections (eTable 1 in the Supplement), excluding visits resulting in symptomatic diagnoses (ie, fever, cough, or diarrhea), recurrent diagnoses within 7 days (ie, repeated treatment or same infection), and diagnoses confirmed as COVID-19. We also excluded patients treated in South Israel who lived elsewhere, owing to a lack of follow-up visits and therefore partial and unreliable data.

### Covariates and Diagnoses

Patient database entries contained patient date of diagnosis, clinic number, visit number, date of birth, sex, residency area, and *ICD-9* diagnosis. Using contiguous *ICD-9* codes and based on anatomical and common clinical classification,^2^ infectious diseases were grouped into 1 of 3 categories: lower respiratory, upper respiratory, or gastrointestinal infections. Non–SARS-CoV-2 respiratory pathogens included detected respiratory viruses (ie, adenovirus, influenza A and B, RSV, parainfluenza, human metapneumovirus, and other human coronaviruses), as well as undefined pathogens, based on clinical diagnosis only. Diagnoses based on only symptoms, such as cough or fever, were excluded. Patients were categorized into 7 age groups based on unique medical and social characteristics and on differences in restrictions that applied to each group (eg, closing daycares, severely restricting social interaction among young children) during and between pandemic lockdowns^2^: 0 to 3 years, 4 to 11 years, 12 to 19 years, 20 to 34 years, 35 to 59 years, 60 to 79 years, and 80 years and older. Visits were categorized by age group and number of insured patients registered with a CHS clinic in the south, then multiplied by 100 000 to obtain age-specific IRs.

### Viral Laboratory Tests

We further examined weekly numbers of respiratory viruses detected by real-time polymerase chain reaction (PCR) using the RV-Essential multiplex assay test (Allplex).^9^ We conducted laboratory investigation only for respiratory infectious diseases, since the tests performed routinely in Israel for gastrointestinal infectious diseases include stool cultures only for common bacteria and not for viruses, which are known to be the most common cause of respiratory and gastrointestinal infections. Tests were analyzed from January 2017 to July 2021 at the largest regional virology laboratory in southern Israel. Numbers and results of nasal swab tests with results positive for respiratory viruses were collected, and proportions were compared each week. Positive results in real-time PCR tests for SARS-CoV-2 were excluded.

### Statistical Analysis

We calculated weekly IRs by infection for each age group from 2017 to 2021 and then compared weekly age and disease-specific rates for April to June each year. We then modeled weekly IRs using interrupted time-series analyses, with variation in time examined as trends and season and cyclical patterns using trigonometrical functions. Given that communicable disease transmission may be associated with weather, weekly maximum temperatures were included in the model. NPIs are associated with decreased seasonal morbidity, so they were included in the model as binary variables to account for intervention and time since the end of each lockdown in the interrupted time-series analyses. To obtain expected weekly IRs during April to June 2021, we fit regression equations for each week based on observed data from previous years (eMethods 1 in the Supplement).

Residual errors from our models could be better explained using autoregressive techniques; however, our intent was to compare current morbidity with morbidity in previous years without relying on proximal observations. Instead, we used daily data from January 1, 2017, to April 1, 2021, to refine the model and then computed expected disease-specific and age-specific IRs for April 1 to June 30, 2021. Actual IRs were divided by expected IRs as estimated by the model to obtain IR ratios (IRRs). A ratio greater than 1.0 indicates an increase in morbidity compared with expected morbidity.

The aforementioned findings would indicate a change in respiratory infectious morbidity compared with previous years. In secondary analyses, we set out to investigate whether a change in pathogens occurred by conducting sensitivity analyses comparing current non–SARS-CoV-2 respiratory infection growth patterns with those observed prelockdown over the same period and at peak season^10^ of previous years. Because most respiratory infections are characterized by droplet and airborne transmission, short incubation time, and low mortality,^10,11^ the ascending phase tends to grow exponentially.^12,13,14^ Using local infectious morbidity surveillance data from the National Center for Infection Control and Antibiotic Resistance,^15^ we first confirmed that winter months (ie, November-January) were characterized by ascending respiratory morbidity and then derived the daily rate of exponential growth (*r*) for each age group using daily cumulative respiratory infections IR,^16^ assuming it equals the number of diagnoses at the beginning of the epidemic multiplied by the exponent of the growth rate^12,13,14^ (eMethods 2 in the Supplement). Natural logs of daily growth rates were compared between years and each period (ie, November-January and April-June), allowing us to examine change in current virulency of infectious agents.^17,18^

The threshold for statistical significance was set a priori at 2-sided *P* < .05. Data analyses were performed using R statistical software version 4.0.3 (R Project for Statistical Computing), including the packages data.table, dplyr, ggplot2 lubridate, and tidyverse.

## Results

### Diagnoses

A total of 1 221 568 visits by 386 711patients across all ages met inclusion criteria. There were 202 494 (52.3%) male patients and 184 217 (47.7%) female patients, and the mean (SD) age was 27.29 (23.93) years. Compared with the April to June COVID-19 lockdown, an increase in mean daily IR for non–SARS-CoV-2 respiratory and gastrointestinal infections was observed in 2021 for all ages (change in IR by age group: 0-3 years, 99.32 [95% CI, 80.33-118.31] infections per 100 000 population; 4-11 years, 19.42 [95% CI, 12.51-26.33] infections per 100 000 population; 12-19 years, 4.92 [95% CI, 2.46-7.38] infections per 100 000 population; 20-34 years, 8.38 [95% CI, 5.39-11.37] infections per 100 000 population; 35-59 years, 9.57 [95% CI, 6.90-12.24] infections per 100 000 population; 60-79 years, 9.32 [95% CI, 6.78-11.87] infections per 100 000 population; ≥80 years, 3.04 [95% CI, 1.37-4.71] infections per 100 000 population) (eTable 2 in the Supplement). Compared with the same months in the prepandemic period, weekly IRs were increased only among children aged 0 to 3 years of age (change in IR, 23.95 [95% CI, 7.02-40.88] infections per 100 000 population; *P* < 001).

### Age-Specific and Disease-Specific Models of Expected Morbidity

**Figure 2.**
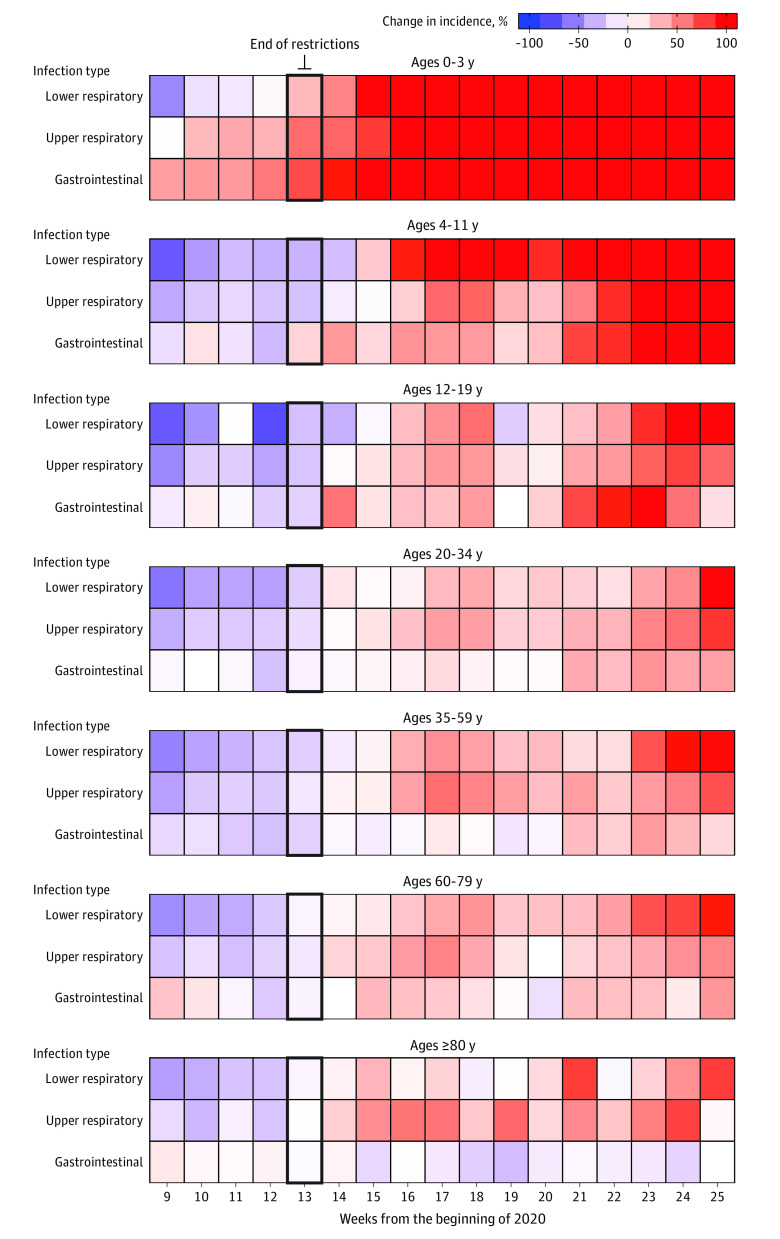
Weekly Infection Incidence Change vs Expected Incidence by Age Group Weekly change in infection incidence for upper and lower respiratory and gastrointestinal disease is given vs expected values based on data since 2017.

**Figure 3.**
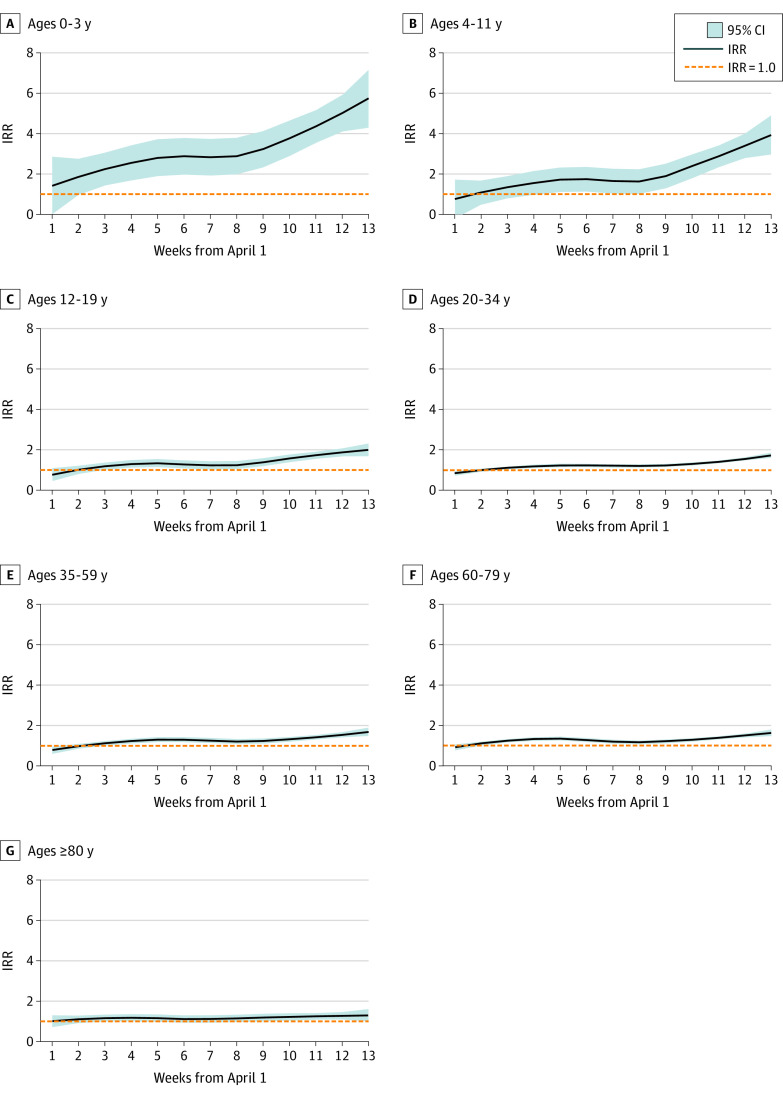
Observed Infectious Rates vs 2017 to 2019 by Age Group Incidence rate ratio (IRR) is presented for observed infectious rates from April to June 2021 vs weekly means from the same periods in 2017 to 2019.

As shown in Figure 2 and Figure 3, April 2021 was a turning point, as the observed IR exceeded expectation for all age groups. The largest ratio between observed and expected IR for the entire period (ie, April-June) was for children aged 0 to 3 years (IRR, 2.64; 95% CI, 2:30-2.91; *P* <.001) (eTable 3 in the Supplement). Individuals aged 4 to 11 years (IRR, 1.57; 95% CI, 1.40-1.71; *P* <.001) and 12 to 19 years (IRR, 1.29; 95% CI, 1.17-1.43; *P* <.001) had smaller differences between observed and expected IRs. Smaller differences were found over this period between expected and observed IRs for individuals aged 20 to 34 years (IRR, 1.25; 95% CI, 1.14-1.33; *P* < .001) and 35 to 59 years (IRR, 1.27; 95% CI, 1.15-1.34; *P* <.001). Upper and lower respiratory infections differed for all ages during April to June 2021 (eTable 4 in the Supplement), exceeding expected weekly IRs. Incidence of all non–SARS-CoV-2 respiratory infections were significantly increased across age groups (IRR, 1.74; 95% CI, 1.56-1.94; *P* < .001). Among children aged 0 to 3 years, the IRR was 2.41 (95% CI, 2.26-3.89; *P* <.001) for upper respiratory infection and 3.74 (95% CI, 2.99-5.21; *P* <.001) for lower respiratory infection. Among older adults (ie, those aged ≥80 years), the IRR was 1.20 (95% CI, 1.06-1.91; *P* = .01) for upper respiratory infection and 1.38 (95% CI, 1.28-2.28; *P* <.001) for lower respiratory infection. Gastrointestinal diseases presented a similar pattern across age groups, except among older adults, for whom the incidence was lower than expected (IRR, 0.90; 95% CI, 0.86-0.92; *P* = .01).

### Respiratory Infection Growth Rate

**Figure 4.**
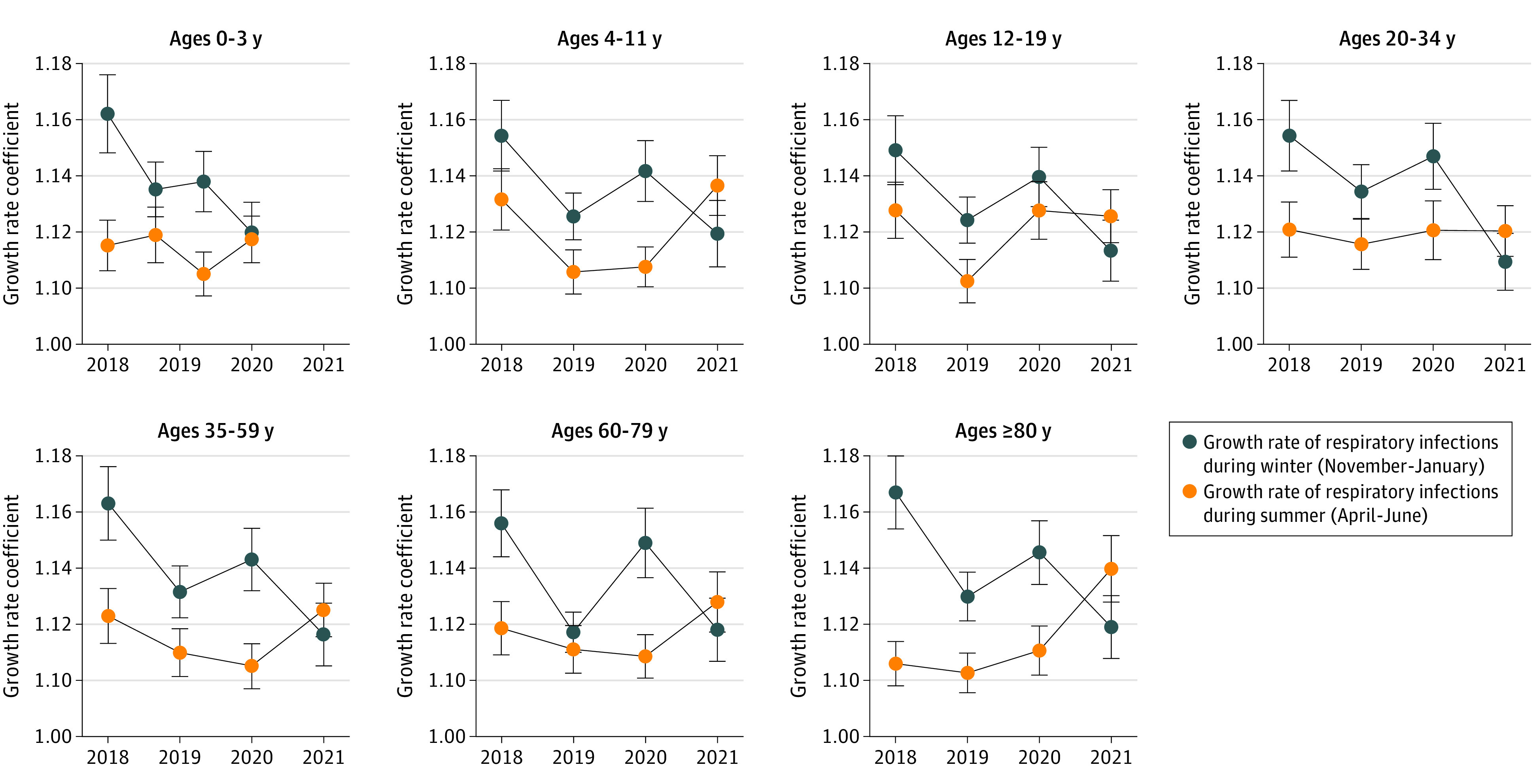
Respiratory Infectious Disease Growth Rates by Season and Age Group Growth rates for respiratory infectious diseases are presented by age group for winter (ie, November-January) and summer (ie, April-June) in 2018 to 2021.

We next examined the growth rates of respiratory infections (Figure 4; eTable 5 in the Supplement). Significant differences were found for older adults in 2021 compared with 2018 to 2019 (change, 0.033; 95% CI, 0.001-0.066; *P* < .001) and 2018 to 2020 (change, 0.037; 95% CI, 0.008-0.058; *P* < .001).

### Laboratory Tests of Respiratory Infections

To investigate associated changes in non–SARS-CoV-2 viral respiratory pathogens from 2018 to 2021, we analyzed 1658 positive real-time PCR nasal swab test results from outpatient visits among individuals of all ages who presented with complicated respiratory symptoms and therefore were admitted to the hospital (eFigure in the Supplement). In contrast to 2018 to 2019, influenza A and B were not detected in 2021. However, a change in relative proportions of positive human metapneumovirus test results was observed in 2018 to 2019, accounting for 58 of 417 tests (13.90%) in 2018 and 41 of 382 tests (10.73%) in 2019, compared with 258 of 798 tests (32.33%) in 2021. No differences were observed in the relative proportions of other non–SARS-CoV-2 viral respiratory pathogens.

## Discussion

This cross-sectional study found that while strict social restrictions from January to April 2021 were associated with significant decreases in infectious disease morbidity (eg, seasonal influenza), the months after the easing of NPIs were associated with the return of non–SARS-CoV-2 respiratory and gastrointestinal infections. This increase in incidence was largest among children aged 0 to 3 years, who presented with the greatest increase in incidence compared with expected rates among all groups examined in this study.

### Host

By the time social restrictions were eased in most countries, individuals aged 0 to 19 years had entered kindergarten or returned to school. Compared with older age groups, this cohort may be less capable of self-hygiene and social hygiene and more susceptible to infection, as suggested by increased IRRs. This increase was observed across disease categories among children aged 0 to 3 years. Differences in morbidity may be explained by various hypothesis regarding immune system development in early life.^19^

Early exposure to a range of microorganisms is associated with strong immune development.^20,21^ However, since March 2020, when lockdowns and restrictions were first implemented in Israel, day care centers, kindergartens, and social infrastructure (eg, parks) have been intermittently closed and reopened. During 3 extended periods of shelter-in-place orders and other social restrictions, young children were restricted to interactions with family within a few familiar settings. It is likely that shelter-in-place orders and prolonged social distancing are associated with adverse outcomes among young children, including increased subsequent susceptibility to pathogens and disease.

Because children are susceptible to infectious diseases, for most illnesses, close surveillance of this group is crucial, especially where schools and day care centers have reopened.^22^ Screening or sample analyses of serological tests and antibodies between cohorts of young children may contribute to forecasting of and preparation for upcoming local epidemics.

Adolescents and adults may be at increased risk for exposure to infectious agents compared with children.^4,22^ Greater exposure is often associated with increased risk of acquiring an infection; however, the primary immune response is particularly affected by aging.

The health of the immune system determines infectious disease mortality and morbidity,^23^ which are relatively high in infancy, low in early childhood, increased somewhat in adulthood, and more steeply increased in later life. Our findings are in accordance with existing epidemiological data, given that we observed that IRRs for most infectious diseases decreased with age. Nevertheless, IRRs were found positively significant for children and young adults aged 4 to 19 years, and we also noticed specific increased respiratory disease morbidity among adults. These findings may be associated with decreased COVID-19 awareness and adherence to social restrictions among children, given that their understanding of hygiene may be limited and they commonly forget to maintain social distance. In contrast, older adults are more prone to isolation^24^ and may be slower to emerge from self-isolation^25^.

### Agents

We found that non–COVID-19 respiratory illnesses significantly increased in April to June 2021 for all age-groups in Israel. In part, this may be associated with increased detection of respiratory viruses (mostly RSV and rhinovirus) as seen in Australia^26^ and Austria^27^ after initial easing of social restrictions. According to a study by Gomez et al,^28^ this atypical late spring increase in respiratory infections may lead to epidemiologic shifts and future epidemics. Similar changes in viral epidemics in other countries may suggest the need for a change in prophylaxis treatment guidelines for populations at increased risk of infection (eg, palivizumab prophylactic antibodies for infants and young children at increased risk for RSV infection).^29^

Our findings suggest an atypical late-spring spike in non–SARS-CoV-2 respiratory infections.^5^ We set out to investigate if this increase significantly differed from the same period in previous years that lacked social restrictions. Given that real-time PCR tests are not routinely performed in community clinics, this trend cannot be explained alone by increased testing. Only for older adults were increases higher than in previous years, suggesting a possible difference in viral reservoir or population susceptibility. Increased rates of human metapneumovirus detected in real-time PCR analyses in 2021 could explain the increase in respiratory infections among older adults; however, in-depth analyses of the pathogens in the current wave of infections are necessary to elucidate possible antigenic and virulence diversity.^30^

### Environment

Environmental exposure and heterologous infections can moderate the effectiveness of the immune system across the lifespan. This may be associated with disparities in morbidity found across the disease groups we examined. Decreases in the incidence of gastrointestinal infections may be associated, in part, with social restrictions imposed and modified during the COVID-19 pandemic. Postponed weddings, canceled flights, and closed parks and restaurants during the pandemic were associated with decreased frequency of multigenerational contacts and prevention of the transmission of diseases via a droplet or fecal-oral route. Changes in health services during the pandemic, some of which decreased in-person availability of services (eg, telemedicine), were also associated with decreased interpersonal contact and, to some extent, transmission of communicable diseases. This study was conducted in a region of Israel where the population is relatively stable, with few demographic changes and consistent health services. Disease mortality and morbidity have been consistent over recent years.

Increased incidence of morbidity from lower and upper respiratory, as well as gastrointestinal, infectious disease among young children, along with an increased rate of growth of non–SARS-CoV-2 respiratory infections among older adults, are of particular concern. In the US, the youngest and oldest patients account for nearly one-third of all emergency room visits.^31^ While populations continue to cope with COVID-19, a resurgence of other communicable diseases may be greater among individuals who consume the most health care resources.

### Limitations

This study has several limitations. Reasons for the observed increase in non–COVID-19 infectious disease morbidity are multifactorial and cannot be fully explained by our findings or routine surveillance data. For instance, we did not measure perceived health or health care–seeking behavior, which may be associated with willingness to engage in community health contact and frequency of doing so. Further research is required to investigate whether people are avoiding clinical contact with respiratory infections for fear of outcomes associated with a COVID-19 diagnosis (eg, possible loss of employment) and further social restrictions.

## Conclusions

This cross-sectional study examined infectious disease incidence rates during 3 months after the cessation of strict social restrictions and a successful vaccination campaign. Overall, we found that this cessation was associated with an increase in non–COVID-19 respiratory infections among all ages examined, with particularly high increases among children ages 0 to 3 years. Morbidity trends observed in Israel in early 2021 may occur in other countries in the coming months, particularly with the ongoing challenges of SARS-CoV-2 variants, and impose additional challenges to health care systems compared with previous years.

## Notes

Supplement. **eTable 1.** Diagnoses and *International Classification of Diseases, Ninth Revision* (*ICD-9*) Codes by Group **eTable 2.** Mean Daily Infectious Disease Morbidity Incidence by Age Group **eTable 3.** Mean Weekly Infectious Disease Morbidity Incidence April to June 2021 vs Expected Rates **eTable 4.** Mean Age-Specific and Disease-Specific Incidence Rates April to June 2021 vs Expected Rates **eTable 5.** Respiratory Infection Mean Seasonal Growth Rate by Age Group 2018 to 2021 **eFigure.** Respiratory Virus Detection by Real-Time Polymerase Chain Reaction
eMethods 1.

eMethods 2.
