# Supplementary material for: Prevalence of Common Infectious Diseases After COVID-19 Vaccination and Easing of Pandemic Restrictions in Israel
Source: JAMA Netw Open. 2022 Feb 1;5(2):e2146175. doi: 10.1001/jamanetworkopen.2021.46175 (PMC8808334; doi:10.1001/jamanetworkopen.2021.46175)
Supplement: Supplement. — eTable 1. Diagnoses and International Classification of Diseases, Ninth Revision (ICD-9) Codes by Group eTable 2. Mean Daily Infectious Disease Morbidity Incidence by Age Group eTable 3. Mean Weekly Infectious Disease Morbidity Incidence April to June 2021 vs Expected Rates eTable 4. Mean Age-Specific and Disease-Specific Incidence Rates April to June 2021 vs Expected Rates eTable 5. Respiratory Infection Mean Seasonal Growth Rate by Age Group 2018 to 2021 eFigure. Respiratory Virus Detection by Real-Time Polymerase Chain Reaction eMethods 1. eMethods 2. [file jamanetwopen-e2146175-s001.pdf]

Supplemental Online Content

Amar S, Avni YS, O'Rourke N, Michael T. Prevalence of common infectious diseases after COVID-19 vaccination and easing of pandemic restrictions in Israel. *JAMA Netw Open*. 2022;5(2):e2146175. doi:10.1001/jamanetworkopen.2021.46175

eTable 1. Diagnoses and *International Classification of Diseases, Ninth Revision* (*ICD-9*) Codes by Group

eTable 2. Mean Daily Infectious Disease Morbidity Incidence by Age Group

eTable 3. Mean Weekly Infectious Disease Morbidity Incidence April to June 2021 vs Expected Rates

eTable 4. Mean Age-Specific and Disease-Specific Incidence Rates April to June 2021 vs Expected Rates

eTable 5. Respiratory Infection Mean Seasonal Growth Rate by Age Group 2018 to 2021

eFigure. Respiratory Virus Detection by Real-Time Polymerase Chain Reaction

eMethods 1.

eMethods 2.

This supplemental material has been provided by the authors to give readers additional information about their work.

© 2022 Amar S et al. *JAMA Network Open*.

eTable 1. Diagnoses and *International Classification of Diseases, Ninth Revision* (*ICD-9*) Codes by Group

Disease Group	Diagnosis	International Classification of Diseases -9 code (ICD-9)
Lower Respiratory Infections	Acute respiratory infections	460-466
	Influenza	487-488
	Pneumonia	480-486
Upper Respiratory Infections	Acute otitis media	3810
	Croup	4644
	Nasal congestion	478
	Pharyngitis\ Streptococcal pharyngitis	0340
	Sinusitis	461
	Upper Respiratory Infections	465,460
	Tonsillitis	463
Gastrointestinal Infections	Gastroenteritis infectious	0091-0092

© 2022 Amar S et al. *JAMA Network Open*.

eTable 2. Mean Daily Infectious Disease Morbidity Incidence by Age Group

Differences between incidence rates of 2021 were compared to daily incidence rates of 2017-2019, and to the daily incidence rates of pandemic year 2020.

	Average daily all-cause morbidity incidence rates (per 100,000) in April-June					Differences between daily incidence rates during April-June 2021 to 2017-2019				Differences between daily incidence rates during April-June 2021 to 2020			
Ages (years)	**2017**	**2018**	**2019**	**2020***	**2021**	**Mean difference**	**CI95% Low**	**CI95% High**	**P-value****	**Mean difference**	**CI95% Low**	**CI95% High**	**P-value****
0-3	121.57	121.18	106.67	41.75	143.43	23.95	7.02	40.88	<0.001	99.32	80.33	118.31	<0.001
4-11	47.29	48.73	43.46	16.53	36.45	-11.05	-17.32	-4.78	<0.001	19.42	12.51	26.33	<0.001
12-19	22.03	21.67	19.77	9.26	14.36	-7.18	-8.65	-5.70	<0.001	4.92	2.46	7.38	<0.001
20-34	32.29	32.68	31.48	16.34	25.20	-7.45	-9.28	-5.62	<0.001	8.38	5.39	11.37	<0.001
35-59	33.14	33.69	34.01	17.83	27.88	-6.31	-8.22	-4.39	<0.001	9.57	6.90	12.24	<0.001
60-79	35.07	34.85	35.00	18.68	28.19	-7.36	-9.89	-4.84	<0.001	9.32	6.78	11.87	<0.001
80+	30.22	26.57	27.38	13.68	16.82	-11.54	-13.31	-9.76	<0.001	3.04	1.37	4.71	<0.001

2020* - this year was characterized by strict limitations and non-pharmaceutical interventions.

P-value** - calculated using the non-parametric paired Wilcoxon test.

© 2022 Amar S et al. *JAMA Network Open*.

eTable 3. Mean Weekly Infectious Disease Morbidity Incidence April to June 2021 vs Expected Rates

Age Group	week (From beginning of April 2021)	Observed weekly Incidence Rate* [Mean±SD]	Predicted weekly mean Incidence Rate	Lower limit of predicted weekly mean Incidence Rate	Higher limit of predicted weekly mean Incidence Rate	Incidence Rate Ratio (IRR)	P-Value**
0-3 Years	1	693.7 ± 558.38	436.24	376.30	505.95	1.59	0.09
	2	712.03 ± 523.4	422.19	370.46	481.30	1.69	0.05
	3	802.56 ± 525.86	406.37	358.57	460.67	1.97	0.04
	4	955.86 ± 663.94	396.81	342.26	460.21	2.41	0.06
	5	1041.39 ± 728.41	385.01	336.48	440.66	2.70	0.06
	6	1049.16 ± 638.8	378.02	324.30	440.80	2.78	0.04
	7	875.32 ± 564.88	368.32	324.07	418.71	2.38	0.04
	8	823.11 ± 530.19	360.83	319.18	408.01	2.28	0.04
	9	988.63 ± 548.49	357.67	316.12	404.76	2.76	0.03
	10	1096.93 ± 661.74	354.70	312.88	402.18	3.09	0.04
	11	1236.34 ± 691.57	338.77	299.88	382.76	3.65	0.04
	12	1297.43 ± 753.84	336.98	294.28	385.96	3.85	0.04
	13	1321.31 ± 708.44	341.95	302.80	386.24	3.86	0.03
4-11 Years	1	144.47 ± 142.77	185.37	158.26	217.18	0.78	0.80
	2	164.79 ± 167.21	171.19	148.82	196.97	0.96	0.61
	3	174.07 ± 175.05	168.75	147.56	193.03	1.03	0.28
	4	209.19 ± 202.71	158.60	135.31	185.95	1.32	0.04
	5	258.9 ± 277.46	156.54	135.44	180.96	1.65	0.11
	6	256.47 ± 265.16	150.97	128.03	178.07	1.70	0.10
	7	209.42 ± 221.58	151.69	132.18	174.12	1.38	0.10
	8	202.57 ± 214.16	152.21	133.39	173.73	1.33	0.09
	9	240.34 ± 249.42	149.98	131.31	171.33	1.60	0.09
	10	291.15 ± 297.51	149.96	131.01	171.68	1.94	0.10
	11	341.29 ± 334.63	135.93	119.19	155.05	2.51	0.10

© 2022 Amar S et al. *JAMA Network Open*.

	12	383.93 ± 363.11	138.57	119.73	160.39	2.77	0.09
	13	366.48 ± 365.64	136.99	120.16	156.19	2.68	0.10
12-19 Years	1	66.52 ± 68.32	89.05	77.07	102.93	0.75	0.88
	2	90.03 ± 85.18	81.45	71.65	92.62	1.11	0.24
	3	90.57 ± 95.49	80.87	71.53	91.46	1.12	0.16
	4	98.58 ± 98.76	75.64	65.40	87.51	1.30	0.12
	5	106.06 ± 109.92	75.12	65.80	85.79	1.41	0.13
	6	105.26 ± 103.15	72.49	62.33	84.33	1.45	0.10
	7	80.42 ± 90.74	73.49	64.79	83.38	1.09	0.26
	8	84.42 ± 84.23	74.37	65.91	83.93	1.14	0.07
	9	106.6 ± 104.03	73.48	65.07	83.00	1.45	0.09
	10	113.28 ± 108.6	73.90	65.32	83.63	1.53	0.09
	11	118.89 ± 110.62	67.78	60.11	76.44	1.75	0.08
	12	123.43 ± 125.55	69.68	60.98	79.63	1.77	0.11
	13	109.27 ± 115.43	68.86	61.09	77.63	1.59	0.13
20-34 Years	1	137.85 ± 112.83	163.03	145.93	182.20	0.85	0.90
	2	155.84 ± 127.92	151.50	137.37	167.12	1.03	0.25
	3	166.62 ± 141.08	150.15	136.70	164.97	1.11	0.15
	4	171.59 ± 150.3	142.02	127.12	158.71	1.21	0.16
	5	188.37 ± 161.5	140.69	127.17	155.69	1.34	0.13
	6	179.3 ± 154.13	136.34	121.54	152.98	1.32	0.15
	7	159.26 ± 130.66	137.05	124.50	150.90	1.16	0.15
	8	162.51 ± 132.02	137.45	125.36	150.74	1.18	0.14
	9	181.01 ± 139.17	135.51	123.51	148.70	1.34	0.08
	10	175.87 ± 137.92	135.24	123.08	148.62	1.30	0.10
	11	191.97 ± 146.22	128.95	117.67	141.34	1.49	0.08
	12	198.48 ± 156.44	130.64	117.99	144.67	1.52	0.10
	13	218.17 ± 168.41	128.91	117.66	141.25	1.69	0.09
35-59 Years	1	156.83 ± 127.2	184.56	164.73	206.84	0.85	0.98
	2	175.73 ± 144.66	172.15	155.68	190.42	1.02	0.39
	3	179.36 ± 140.99	168.85	153.35	185.96	1.06	0.23
	4	208.1 ± 175.6	159.51	142.36	178.78	1.30	0.16
	5	231.39 ± 195.3	156.48	141.09	173.60	1.48	0.13

© 2022 Amar S et al. *JAMA Network Open*.

	6	210.52 ± 176.93	150.78	134.04	169.68	1.40	0.14
	7	191.92 ± 168.35	149.92	135.90	165.43	1.28	0.19
	8	181.48 ± 144.65	148.88	135.52	163.60	1.22	0.16
	9	197.36 ± 153.49	145.87	132.71	160.37	1.35	0.11
	10	177.55 ± 127.52	144.53	131.31	159.12	1.23	0.09
	11	200.39 ± 125.1	136.22	124.10	149.54	1.47	0.04
	12	211.27 ± 137.25	137.02	123.55	151.99	1.54	0.06
	13	216.57 ± 160.74	134.89	122.94	148.02	1.61	0.10
60-79 Years	1	173.36 ± 74.11	189.26	169.60	211.33	0.92	0.95
	2	199.78 ± 91.75	179.77	163.14	198.18	1.11	0.14
	3	212. ± 75.29	174.64	159.19	191.68	1.21	0.03
	4	223.22 ± 88.97	166.90	149.61	186.28	1.34	0.05
	5	229.5 ± 95.9	162.39	147.05	179.42	1.41	0.05
	6	212.99 ± 83.87	156.87	140.16	175.67	1.36	0.05
	7	178.65 ± 62.34	153.95	140.18	169.14	1.16	0.08
	8	160.81 ± 61.23	151.21	138.27	165.43	1.06	0.32
	9	185.58 ± 44.64	147.82	135.13	161.77	1.26	0.00
	10	192.51 ± 49.69	145.50	132.86	159.41	1.32	0.01
	11	197.14 ± 50.27	136.55	125.08	149.13	1.44	0.03
	12	197.8 ± 68.44	135.89	123.29	149.83	1.46	0.06
	13	216.29 ± 50.13	134.04	122.86	146.29	1.61	0.02
80+ Years	1	113.95 ± 70.22	119.29	105.64	134.71	0.96	0.86
	2	127.82 ± 73.9	114.85	103.08	127.96	1.11	0.04
	3	139.51 ± 95.12	111.62	100.64	123.80	1.25	0.16
	4	127.82 ± 57.32	107.84	95.37	121.94	1.19	0.13
	5	128.55 ± 66.33	105.13	94.00	117.58	1.22	0.14
	6	98.61 ± 41.81	102.26	90.01	116.18	0.96	0.61
	7	108.83 ± 47.82	100.45	90.32	111.72	1.08	0.36
	8	109.56 ± 49.53	98.93	89.35	109.53	1.11	0.20
	9	142.43 ± 84.53	97.25	87.77	107.75	1.46	0.15
	10	97.88 ± 21.95	96.13	86.63	106.68	1.02	0.43
	11	107.37 ± 29.64	90.07	81.40	99.67	1.19	0.17
	12	118.33 ± 43.83	89.97	80.41	100.67	1.32	0.17

© 2022 Amar S et al. *JAMA Network Open*.

	13	118.33 ± 63.4	89.12	80.57	98.57	1.33	0.20

Incidence rate* - was calculated as number of diagnoses divided by number of patients at the same age group multiplied by 100,000.

P-value** - calculated using the non-parametric paired Wilcoxon test.

© 2022 Amar S et al. *JAMA Network Open*.

eTable 4. Mean Age-Specific and Disease-Specific Incidence Rates April to June 2021 vs Expected Rates

Age Group	Infectious disease	Observed weekly Incidence Rate* [Mean±SD]	Predicted weekly mean Incidence Rate	Lower limit of predicted weekly mean Incidence Rate	Higher limit of predicted weekly mean Incidence Rate	Incidence Rate Ratio (IRR)	P-Value**
0-3 Years	Upper Respiratory Infections	1660.58 ± 289.9	687.82	606.68	779.89	2.41	0.00
	Lower Respiratory Infections	435.14 ± 144.82	116.22	96.96	139.34	3.74	0.00
	Gastrointestinal Infections	879.77 ± 217.29	323.00	283.49	368.05	2.72	0.00
4-11 Years	Upper Respiratory Infections	539.7 ± 162.37	352.89	307.00	405.70	1.53	0.01
	Lower Respiratory Infections	91.81 ± 42.35	37.88	31.31	45.84	2.42	0.00
	Gastrointestinal Infections	116.89 ± 30.26	72.32	63.32	82.62	1.62	0.00
12-19 Years	Upper Respiratory Infections	212.21 ± 33.01	162.50	143.64	183.86	1.31	0.00
	Lower Respiratory Infections	23 ± 6.05	17.69	14.65	21.36	1.30	0.07
	Gastrointestinal Infections	63.26 ± 16.79	45.08	39.49	51.46	1.40	0.00
20-34 Years	Upper Respiratory Infections	332.47 ± 34.75	255.98	233.02	281.20	1.30	0.00
	Lower Respiratory Infections	54.97 ± 6.8	45.17	39.12	52.15	1.22	0.01
	Gastrointestinal Infections	140.29 ± 31.91	118.28	107.62	130.00	1.19	0.01
35-59 Years	Upper Respiratory Infections	370.01 ± 42.77	276.23	251.23	303.73	1.34	0.00
	Lower Respiratory Infections	107.95 ± 14.57	83.98	73.27	96.26	1.29	0.01
	Gastrointestinal	107.84 ± 26.77	98.95	90.26	108.48	1.09	0.27

© 2022 Amar S et al. *JAMA Network Open*.

	Infections						
60-79 Years	Upper Respiratory Infections	254.14 ± 35.13	199.82	183.92	217.10	1.27	0.00
	Lower Respiratory Infections	218.55 ± 19.86	165.82	146.15	188.15	1.32	0.00
	Gastrointestinal Infections	122.61 ± 22.4	103.93	96.02	112.50	1.18	0.01
80+ Years	Upper Respiratory Infections	100.8 ± 16.93	72.79	65.42	81.00	1.38	0.00
	Lower Respiratory Infections	181.03 ± 38.03	151.19	134.68	169.75	1.20	0.01
	Gastrointestinal Infections	73.32 ± 9.87	81.30	73.41	90.04	0.90	0.01

Incidence rate* - was calculated as number of diagnoses divided by number of patients at the same age group multiplied by 100,000.

P-value** - calculated using the non-parametric paired Wilcoxon test.

© 2022 Amar S et al. *JAMA Network Open*.

eTable 5. Respiratory Infection Mean Seasonal Growth Rate by Age Group 2018 to 2021

		Mean seasonal infectious growth rate (r)					
Age Group	**Season**	**2018**	**2019**	**2020**	**2021**	**P-Value*** Significance of differences between growth rates in 2021 compared to 2018-2020	**P-Value*** Significance of differences between growth rates in 2021 compared to 2018-2019
0-3 Years	Winter (Nov-Jan)	1.16 (1.05 - 1.27)	1.14 (1.06 - 1.22)	1.14 (1.05 - 1.23)	1.12 (1.03 - 1.21)	0.015	0.014
	Summer (April-June)	1.12 (1.03 - 1.21)	1.12 (1.03 - 1.21)	1.11 (1.04 - 1.18)	1.12 (1.04 - 1.2)	0.855	0.901
4-11 Years	Winter (Nov-Jan)	1.15 (1.05 - 1.25)	1.13 (1.07 - 1.19)	1.14 (1.05 - 1.23)	1.12 (1.03 - 1.21)	0.075	0.045
	Summer (April-June)	1.13 (1.03 - 1.23)	1.11 (1.04 - 1.18)	1.11 (1.04 - 1.18)	1.14 (1.04 - 1.24)	0.692	0.435
12-19 Years	Winter (Nov-Jan)	1.15 (1.05 - 1.25)	1.12 (1.06 - 1.18)	1.14 (1.06 - 1.22)	1.11 (1.02 - 1.2)	0.053	0.025
	Summer (April-June)	1.13 (1.03 - 1.23)	1.1 (1.03 - 1.17)	1.13 (1.03 - 1.23)	1.13 (1.04 - 1.22)	0.656	0.805
20-34 Years	Winter (Nov-Jan)	1.15 (1.05 - 1.25)	1.13 (1.06 - 1.2)	1.15 (1.06 - 1.24)	1.11 (1.03 - 1.19)	0.014	0.003
	Summer (April-June)	1.12 (1.03 - 1.21)	1.12 (1.03 - 1.21)	1.12 (1.02 - 1.22)	1.12 (1.03 - 1.21)	0.935	0.964
35-59 Years	Winter (Nov-Jan)	1.16 (1.06 - 1.26)	1.13 (1.06 - 1.2)	1.14 (1.05 - 1.23)	1.12 (1.03 - 1.21)	0.011	0.003
	Summer (April-June)	1.12 (1.03 - 1.21)	1.11 (1.03 - 1.19)	1.11 (1.03 - 1.19)	1.13 (1.04 - 1.22)	0.967	0.695
60-79 Years	Winter (Nov-Jan)	1.16 (1.07 - 1.25)	1.12 (1.06 - 1.18)	1.15 (1.05 - 1.25)	1.12 (1.03 - 1.21)	0.098	0.02
	Summer (April-June)	1.12 (1.03 - 1.21)	1.11 (1.03 - 1.19)	1.11 (1.04 - 1.18)	1.13 (1.03 - 1.23)	0.534	0.384
80+ Years	Winter (Nov-Jan)	1.17 (1.07 - 1.27)	1.13 (1.06 - 1.2)	1.15 (1.06 - 1.24)	1.12 (1.03 - 1.21)	0.011	0.005
	Summer (April-June)	1.11 (1.03 - 1.19)	1.1 (1.03 - 1.17)	1.11 (1.03 - 1.19)	1.14 (1.03 - 1.25)	< 0.001	<0.001

P-value** - calculated using Analysis of variance test (ANOVA).

© 2022 Amar S et al. *JAMA Network Open*.

eFigure. Respiratory Virus Detection by Real-Time Polymerase Chain Reaction

Viruses detected by RT-PCR from nasal swabs specimens obtained from patients with respiratory tracts infections between April-June, 2018-2021. N equals total tests performed per period, with the positive percentage (pos%) below for each year ; bars reflect proportions (%) of each virus detected from the total number of positive tests performed.

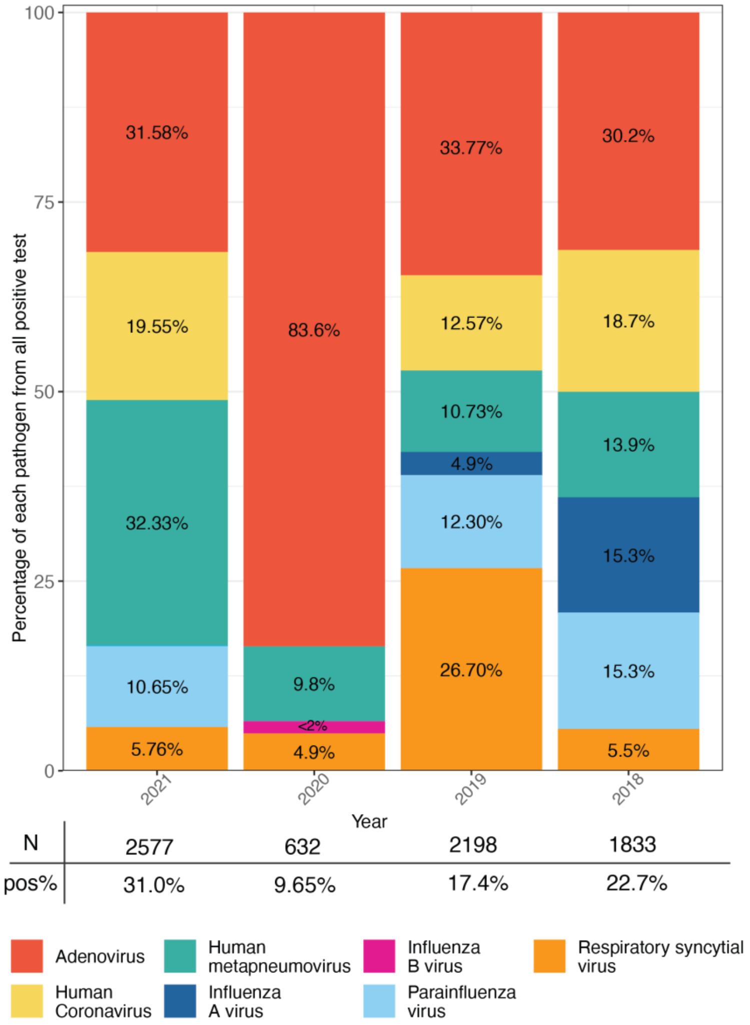

© 2022 Amar S et al. *JAMA Network Open*.

eMethods 1.

Formula- estimating weekly incidence rates using linear regression model, with variation in time examined as trends, season and cyclical patterns, using trigonometrical functions.

$$\log(Incidence) = \beta_0 + \beta_1(week_i) + \beta_2(\sin(2\pi\frac{week_i}{52})) + \beta_3(\cos(2\pi\frac{week_i}{52})) + \beta_4(season) + \beta_5\left(\max temp_{week_i}\right) + \beta_6(lockdown_2) + \beta_7\left(week_i - week_{lockdown_2}\right) + \beta_8(lockdown_3) + \beta_9\left(week_i - week_{lockdown_3}\right)$$

$Week_i$ – Number of week since the beginning of the year 2021; Season – calendric seasons based on national definition (winter, spring, summer, autumn): $lockdown_2$ and $lockdown_3$ - binary variables representing existence of lockdown in $week_i$ (intervention). $week_i$-$week_{lockdown}$ – the time (in weeks) passed since beginning of each lockdown (intervention).

© 2022 Amar S et al. *JAMA Network Open*.

eMethods 2.

Formula- calculating daily rate of exponential growth for each age-group using the daily cumulative respiratory infections incidence rate (C_{IR}).

$$C_{IR}(t_i)_{j,p,y} = C_{IR}(0)_{j,p,y}e^{r_{j,p,y}t_i}$$

cumulative IR (C_{IR}) of each group (j) in year (y) and period (p) at time t_i, equals to the number of cases at the start of epidemic ($C_{IR}(0)$), multiplied by exponent of the growth rate (r) constant and time; t_i: a day in calendric year; (j): age group: 0-3, 4-11, 12-19,20-34,35-59,60-79,80+; (p): seasonal period: November-January or April-June; (y) year between 2018-2021; (r): growth rate constant.

© 2022 Amar S et al. *JAMA Network Open*.
